# Is time of birth a predictor of adverse perinatal outcome? A hospital-based cross-sectional study in a low-resource setting, Tanzania

**DOI:** 10.1186/s12884-017-1358-9

**Published:** 2017-06-12

**Authors:** Andrew Mgaya, Januarius Hinju, Hussein Kidanto

**Affiliations:** 1 Department of Obstetrics and Gynaecology, Muhimbili National Hospital, P.O. Box 65000, Dar es Salaam, Tanzania; 2 Department of Women’s and Children’s Health, International Maternal and Child Health, Academic Hospital, Uppsala, Sweden; 3 Department of Obstetrics and Gynaecology, Benjamin Mkapa referral Hospital, Dodoma, Tanzania; 4 Department of Obstetrics and Gynaecology, Muhimbili University of Health and Allied Sciences, Dar es Salaam, Tanzania

**Keywords:** Perinatal outcome, Time of birth, Quality of care, Low-resource setting

## Abstract

**Background:**

Inconsistent evidence of a higher risk of adverse perinatal outcomes during off-hours compared to office hours necessitated a search for clear evidence of an association between time of birth and adverse perinatal outcomes.

**Methods:**

A cross-sectional study conducted at a tertiary referral hospital compared perinatal outcomes across three working shifts over 24 h. A checklist and a questionnaire were used to record parturients’ socio-demographic and obstetric characteristics, mode of delivery and perinatal outcomes, including 5th minute Apgar score, and early neonatal mortality. Risks of adverse outcomes included maternal age, parity, referral status and mode of delivery, and were assessed for their association with time of delivery and prevalence of fresh stillbirth as a proxy for poor perinatal outcome at a significance level of *p* = 0.05.

**Results:**

Off-hour deliveries were nearly twice as likely to occur during the night shift (odds ratio (OR), 1.62; 95% confidence interval (CI), 1.50–1.72), but were unlikely during the evening shift (OR, 0.58; 95% CI, 0.45–0.71) (all *p* < 0.001). Neonatal distress (O.R, 1.48, 95% CI; 1.07–2.04, *p* = 0.02), early neonatal deaths (OR, 1.70; 95% CI, 1.07–2.72, *p* = 0.03) and fresh stillbirths (OR, 1.95; 95% CI, 1.31–2.90, *p* = 0.001) were more significantly associated with deliveries occurring during night shifts compared to evening and morning shifts. However, fresh stillbirths occurring during the night shift were independently associated with antenatal admission from clinics or wards, referral from another hospital, and abnormal breech delivery (OR 1.9; 95% CI, 1.3–2.9, *p* = 0.001, for fresh stillbirths; OR, 5.0; 95% CI 1.7–8.3, *p* < 0.001, for antenatal admission; OR, 95% CI, 1.1–2.9, *p* < 0.001, for referral form another hospital; and OR 1.6; 95% CI 1.02–2.6, *p* = 0.004, for abnormal breech deliveries).

**Conclusion:**

Off-hours deliveries, particularly during the night shift, were significantly associated with higher proportions of adverse perinatal outcomes, including low Apgar score, early neonatal death and fresh stillbirth, compared to morning and evening shifts. Labour room admissions from antenatal wards, referrals from another hospital and abnormal breech delivery were independent risk factors for poor perinatal outcome, particularly fresh stillbirths.

## Background

Improvement of the quality of obstetric and neonatal care is regarded as a prerequisite for preventing maternal and perinatal mortality [1], especially in the post-millennium development goal era [2]. In low-resource settings, substandard care accounts for significant numbers of maternal [3] and neonatal deaths [4], of which one-third include intrapartum stillbirth [5]. In Tanzania, substandard care contributes to 6% of labour-related maternal deaths [6] and 30% of perinatal mortality due to intrapartum asphyxia of mostly term deliveries [7]. Numerous studies have suggested that there is a higher risk of adverse perinatal outcome during off-hours deliveries, i.e., during evening and night shifts, or at weekends [8–15], compared to morning and afternoon office hours. These adverse outcomes include fetal injury [9, 11], perinatal mortality [8, 10] and early neonatal death (ENND) [12, 13]. Other studies associated intrapartum fetal death [13], and admission to neonatal intensive care unit (NICU) [9], with births occurring during weekends. Conversely, other authors did not find any difference in the risk of poor perinatal outcome during off-hours [16–18]. This evidence of high risk of adverse outcomes during off-hours has led to a general questioning of the quality of care and availability of access to skilled care providers, organization of work and availability of equipment, and supplies during off-hours.

One study, reporting on the circadian rhythm of the perinatal mortality rate throughout the week, found that the strongest determinants of the rate were associated with the selection of time of delivery of low birth weight fetuses in the afternoon hours based on obstetric risks, availability of obstetric and neonatal care, and chronobiologic behaviour [19]. Another author associated increased adverse perinatal outcomes with increased rate of low umbilical artery pH in caesarean section (CS) during night shifts [20]. However, umbilical blood gas analysis has also been found to be unreliable as a neonatal assessment tool for infants with an Apgar score of less than 7; and especially in high-risk pregnancies, including those delivered by CS, pre-term pregnancies and twin pregnancies [21].

Stress and severe fatigue in care professionals that is related to long working hours compromises patients’ safety [22–24]; however, the link between hours of work and patient safety is still unclear due to a lack of consistent reports of an association of sleep deprivation with medical error [25, 26], and patient safety [27]. Nonetheless, restricted on-call duty (50–80 working hours per week) has been reported to alleviate acute stress, fatigue, and loss of sleep [28, 29]; and also has a positive impact on the quality of life of health care providers [30], and, subsequently, can improve quality of care [2]. Coincidental observations through attending hospital management and departmental meetings at Muhimbili National Hospital (MNH) highlighted low patient-to-caregiver ratio, periodic unanticipated high rates of deliveries, and limited supplies during night and evening shifts compared to morning shifts. Despite these discussions, no scientific evidence has been sought to confirm how the discrepancy between the demand and supply of obstetric care affects pregnancy outcomes.

Few researchers in developing countries [31], and none we know of in Tanzania, have addressed the relationship between time of delivery and perinatal outcome. Lack of clear evidence of whether quality of care differs between time of delivery in relation to the structure and process of care compromises the overall effectiveness and efficiency of obstetric and neonatal interventions. Understanding the contributing factors for perinatal morbidities and mortalities is the key to reducing the number of adverse perinatal outcomes. Therefore, this study intended to answer the following research question: Is time of birth a predictor of adverse perinatal outcome? We estimated and compared the prevalence of adverse perinatal outcome across the three hospital working shifts, including the morning shift (7 am–2 pm), evening shift (2 pm–8 pm), and night shift (8 pm–7 am).

## Methods

### Study design and settings

We conducted a cross-sectional study at MNH in 2012. MNH is the highest referral hospital in Tanzania, and is a teaching hospital for Muhimbili University of Health and Allied Sciences (MUHAS). The hospital is situated in Dar es Salaam, which, according to the Tanzania Demographic Health Survey (TDHS 2010), has a population of about 4.5 million and an annual population growth rate of 4.3%. As one of the four consultant hospitals in Tanzania, MNH serves as a referral centre for the city of Dar es Salaam and the neighbouring coastal region. The annual number of deliveries in the hospital in 2012 was between about 8000 and 9000, of which 70% were self-referrals and hence the majority were also low-risk deliveries. Furthermore, out of 15 to 40 daily deliveries, 30 to 60% delivered by CS [32]. The peak delivery period was March to April and the lowest delivery period was December to January. According to the birth registry, during 2012–2015, the MMR of women who delivered at MNH increased from 96 to 146/100,000 live births, and the fresh stillbirth ratio and early neonatal deaths decreased from 32/1000 to 26/1000 and from 27/1000 to 16/1000, respectively.

The obstetric wards were attended by 35 obstetricians, 25 obstetrics and gynecology residents, 2 registrars and 25 nurse midwives. Daily routines included a daily grand ward round for 24-h call reports, and major ward rounds in the labour room, obstetric ICU and admitting antenatal/postnatal ward. The outpatients’ clinic and elective obstetrics and gynecological surgeries were included among daily hospital routines. Doctors on call work for 24 h in a team comprised of one intern doctor, two residents (one in the labour ward and another in the obstetric theater), and one specialist and one consultant obstetrician who were mostly called for a second opinion or in case of emergency. The length of working hours for nurses was 8 h on the morning, 6 h on the evening and 11 h on the night shift. The nurse-parturient ratio averaged at 1:3–5 on the morning and evening shifts, but 1:6–8 on the night shifts. After a normal vaginal delivery, the mothers and babies were observed in hospital for 6–10 h. During this time, babies are vaccinated with BCG and polio vaccinations before being discharged. Babies delivered by CS or those who were sick were admitted to the neonatal ward, which also admits sick babies from other lower referral hospitals.

### Study population and sampling

All parturients who delivered at MNH during the study period were identified from the midwifery registry and assessed for eligibility for recruitment. The exclusion criteria included patients that had a high risk of, or preexisting conditions that could lead to, poor maternal and/or perinatal outcomes: history of peripartum complication such as abruption placenta, placenta previa and eclampsia; and neonatal complications, including congenital anomalies and intrauterine fetal deaths confirmed antenatally by ultrasound or macerated stillbirths. All parturients who delivered <28 weeks of gestation, those who delivered twins, and those delivered by elective CS were also excluded.

### Data collection

Data were collected for 5 months (June to December) from the partogram and case notes using a checklist and questionnaire. The variables of interest included: socio-demographic characteristics, such as age, marital status and level of education; obstetric characteristics, including parity, gestation age, source of admission, number of gestations, nature of labour and mode of delivery; and perinatal outcomes, including 5th minute Apgar scores, birth trauma, intrapartum stillbirth and early neonatal death. The data were collected by one of the researchers from the midwifery registry, however, in case of incomplete or ambiguous information from the registry, mothers were interviewed for clarification. Neonates who were admitted to the neonatal unit were followed up for seven days to document the early neonatal outcome. Alternatively, neonates who were not admitted after delivery were considered to have good outcomes, hence they were not followed up. Verification of the information recorded by questionnaire and checklist was completed daily, and hence, no case was excluded from the analysis.

### Definition of terms

*Adverse perinatal outcomes* were defined as; Apgar score < 7 at 5 min, fresh stillbirth, early neonatal death, and physical birth trauma (including skull fracture, clavicle fracture, facial nerve palsy and brachial plexus injuries). The *perinatal period* commenced from 28 completed weeks of gestation or when the neonatal birth weight was above 500 g.

*Early neonatal death* was defined as death of a newborn within 7 days post-delivery. A *stillbirth* was defined as the delivery of a dead newborn within the perinatal period, and *fresh stillbirth* was the birth of a dead fetus without postmortem changes based on physical appearance, including blistered or peeled-off skin. A *perinatal death* was a neonatal death during the perinatal period and hence included stillbirths and early neonatal deaths. *Primipara* referred to a woman experiencing her first delivery, while *Multipara* was defined as a woman having her second or more delivery. A *work shift* meant a period of working hours within a 24-h cycle that was divided into a *morning shift* from 7 am to 2 pm (8 h), an e*vening shift* from 2 pm to 7 pm (6 h), and a *night shift* from 7 pm to 7 am (12 h).

### Data analysis

Data entry and cleaning was performed using Epi Info™ version 6, and then data were transferred to SPSS version 21.0 (SPSS, Armonk, NY: IBM Corporation) for statistical analyses. Data cleaning included removing typographic errors and duplicated information and amending incomplete or incongruent information using case notes and ward report logs and by interviewing the mother. Statistically significant differences in associations between maternal characteristics, mode of delivery and perinatal outcome to time of birth were assessed using chi-squared χ^2^ test for categorical variables, while Student’s *t*-test was used to assess continuous variables at a significance level of *p* = 0.05. Multiple logistic regression analysis was used to measure independent associations between selected predictors of adverse outcomes in relation to night shifts.

## Results

During the study period, 3193 women delivered, out of which 557 women were excluded. Excluded participants were 327 who delivered by elective CS, and 9 with multiple gestations, of which 7 were twins and 2 were triplets. Other excluded women were 49 who had abruption placentas, and 99 who delivered before 28 weeks of gestation. One mother who had anencephalic fetus, 51 who had macerated stillbirths, and 22 with antenatal sonographic diagnosis of intra-uterine fetal deaths (IUFD), were also excluded. Therefore, the remaining 2636 deliveries were analysed (Table 1). Most of the deliveries occurred during the night shift (49%), and the evening shift had the least number of deliveries (21%). Thus, off-hours deliveries were nearly twice as likely to occur during the night shift (OR, 1.62; 95% CI, 1.50–1.72, *p* < 0.001) but were less likely during the evening shift (OR, 0.58; 95% CI, 0.45–0.71, *p* < 0.001).

**Table 1:** Percentage distribution of deliveries in three shifts within 24 h

Time of delivery	No. of deliveries *N* = 2636(%)	OR(95%CI)	*P*-value
Morning shift	780 (30.0%)	1	
Evening shift	577 (21.0%)	0.58 (0.45–0.71)	<0.001
Night shift	1279 (49.0%)	1.62(1.50–1.72)	<0.001

When comparing obstetric characteristics to timing of delivery (Table 2), the majority of deliveries were parturients aged between 20 and 35 years (80%), with a primary school education (60%) and were multiparas (65%). Furthermore, almost all deliveries were full-term delivery (92%). Most teenagers delivered during the morning shift (7.2%) compared to the evening (3.9%) and night shifts (2.3%). Parturients aged 35 years and above were nearly evenly distributed during the morning shift (14.1%) compared to the evening (13.5%) and night shifts (13.8%). Nearly the whole studied group had some level of formal education, i.e., at least primary education level, with the majority being multiparas and evenly distributed across the morning (63.5%), evening (68.3%) and night shifts (64.2%). Two-thirds of the deliveries came direct to the hospital from home, and nearly all of the remainder were referred from other hospitals. Referrals from other hospitals mostly delivered during the night shift (38.2%) compared to evening (33.3%) and morning shifts (29.9%). On the other hand, parturients arriving from home mostly delivered during the morning shift (68.7%) compared to those who delivered in the evening (65%) and night shifts (60.8%).

**Table 2:** Demographic and obstetric characteristics of according to time of birth

Socio-demographic characteristics	Time of delivery (Shifts)		
	Morning *n* = 780(%)	Evening *n* = 577(%)	Night *n* = 1279(%)
Age			
< 20 yrs	56 (7.2)	23 (3.9)	29 (2.3)
20-35 yrs	614 (78.8)	476 (82.5)	1012 (83.8)
35 + yrs	110 (14.1)	78 (13.5)	176 (13.8)
Education level			
No formal education	10 (1.3)	13 (2.2)	19 (1.5)
Primary School	443 (56.8)	338 (58.6)	792 (62.0)
Secondary School	216 (27.7)	146 (25.3)	305 (23.8)
College education	111 (14.2)	80 (13.9)	163 (12.7)
Parity			
Primipara	276 (35.4)	192 (33.3)	458 (35.8)
Multipara	504 (64.6)	385 (66.7)	821 (64.2)
Gestation age			
36 weeks	76 (9.7)	48 (8.3)	106 (8.3)
37 + weeks	704 (90.3)	529 (91.7)	1173 (91.7)
Source of admission			
Referral another Hospital	233 (29.9)	192 (33.3)	489 (38.2)
From home	536 (68.7)	375 (65.0)	777 (60.8)
Antenatal admissions	11 (1.4)	109 (1.7)	6 (1.0)

When comparing the distribution of mode of deliveries (Table 3), half of the studied group delivered vaginally, and the majority of the remaining half by cesarean section (46%). Equal distribution of the remaining 4% of participants had abnormal breech delivery or were delivered by low cavity vacuum extraction (LCVE). No statistically significant difference was found in the rate of vaginal deliveries across the three shifts, i.e., the morning (52%), evening (52%) and night shifts (48%). Similarly, CS deliveries were evenly distributed during the morning (46%), evening (47%) and night shifts (48%) (*p* = 0.07). However, most abnormal breech deliveries (1.6%) occurred during the night shift compared to morning (1%) and evening shifts (0.5%) (*p* = 0.02). A similar pattern was observed for the LCVE deliveries, most of which (2.3%) were performed at night compared to the morning (1.8%) and evening (0.9%) shifts, (*p* = 0.03)

**Table 3:** Percentage distribution of mode of delivery in three shifts within 24 h

Mode of delivery	Time of delivery (Shifts)			*P*-value
	Morning *n* = 780(%)	Evening *n* = 577(%)	Night *n* = 1279(%)	
SVD	397 (51.0)	300 (52.0)	623 (48.6)	0.1
C/S	361 (46.2)	269 (46.6)	606 (47.5)	0.07
ABD	8 (1.0)	3 (0.5)	21 (1.6)	0.02
LCVE	14 (1.8)	5 (0.9)	29(2.3)	0.03

Poor perinatal outcome was associated with 10% of neonatal distress (Apgar score < 7 at 5th min); 4% of early neonatal deaths and 6% of fresh stillbirths (Table 4). Night shift deliveries had twice the odds of poor perinatal outcome compared to morning shift deliveries (OR 1.48, 95% CI; 1.07–2.04, *p* = 0.02, for neonatal distress; OR, 1.70; 95% CI, 1.07–2.72, *p* = 0.03, for ENND; and OR, 1.95; 95% CI, 1.31–2.90, *p* = 0.001, for FSB). No significant difference was found in the odds of poor perinatal outcome in the evening shift compared to the morning shift deliveries. Using multiple logistic regression analysis (Table 5), night shifts were independently associated with fresh stillbirths, antenatal admission from clinic or antenatal wards, referral from another hospital, and abnormal breech delivery (OR 1.9; 95% CI, 1.3–2.9; *p* = 0.001, for FSB; OR 5.0; 95% CI 1.7–8.3; *p* < 0.001, for antenatal admission; OR 1.2; 95% CI, 1.1–2.9; *p* < 0.001, for referral from another hospital; and OR 1.6: 95% CI 1.02–2.6; *p* = 0.004, for abnormal breech delivery(ABD)).

**Table 4:** Perinatal outcome of current delivery according to three shifts of 24 h

Perinatal outcome	Time of delivery (Shifts)							
	Morning		Evening			Night		
	*N* = 780(%)	OR (95%CI)	*N* = 577(%)	OR(95%CI)	*P*-Value	*N* = 1279(%)	OR(95%CI)	*P*-value
Apgar Score < 7 at 5^th^min	57 (7.3)	1	44 (7.6)	1.05 (0.09–1.58)	0.83	134 (10.4)	1.48 (1.07–2.04)	0.02
END	25 (3.2)	1	18 (3.1)	0.97 (0.53–1.79)	0.93	69 (5.3)	1.70 (1.07–2.72)	0.03
FSB	35 (4.4)	1	29 (5.01)	1.16 (0.69–1.93)	0.65	105 (8.2%)	1.95 (1.31–2.90)	0.001

**Table 5:** Logistic regression of predictors of adverse outcome associated with the night shift

Variable	OR (95% CI)	*P*-value
Fresh still birth	1.9 (1.3–2.9)	0.001
Maternal Age		
< 20 yrs	1.0 (0.9–2.9)	0.98
> 35 yrs	1.0 (0.9–2.8)	0.76
Primiparity	1.0 (0.9–1.9)	0.87
Antenatal admission	5.0 (1.7–8.3)	<0.001
Referrals from another Hospital	1.2 (1.1–2.9)	<0.001
Mode of delivery		
SVD	1.0 (0.9–2.7)	0.1
LCVE	1.3 (0.9–3.3)	0.21
ABD	1.6 (1.02–2.6)	0.04

## Discussion

### Main findings

The night shift was significantly associated with adverse perinatal outcomes, including low Apgar score, early neonatal death, and fresh stillbirth when compared with the morning and evening shifts. Our findings were consistent with some previous findings [10, 12, 13]; although refuted by others [17, 18] from settings with access to standardized care. The Dar es Salaam public hospitals birth registries, including that of MNH, confirmed that most of the daily deliveries occurred during the night shift compared to the morning and evening shifts. [32–35]. The most common groups of patients who were admitted to the delivery room during the night shift included patients from antenatal wards, referrals from other hospitals, and a significant number were abnormal breech deliveries. These groups of patients presumably possessed an independent risk of fresh stillbirth that was associated with maternal and fetal characteristics or obstetric risk factors that either led to their admission from the antenatal care or caused by delays in care as they travelled through the referral system. Therefore, the demonstrated risk of poor perinatal outcomes during off-hours in this study could have been associated with timing of delivery rather than the quality of care provided during off-hours. Alternatively, the exclusion of parturients with a known history of pregnancy complications to eliminate the confounding effect of antenatal complication might have reduced the resulting proportions of adverse perinatal outcome, either among antenatal admissions or referred patients.

In our study, teenagers were not associated with adverse perinatal outcomes and the majority delivered in the morning shift. However, teenage pregnancy has been found to have higher perinatal risks, including anaemia [36], placental problems [37], preterm delivery [36–38] and low birth weight [15]. Nevertheless, although not part of this study, teenagers require special attention due to the above-mentioned risks and others, including adherence to antenatal care and late booking [39], substance abuse [40], and possibly financial instability and dependence compared to adult and/or married pregnant women [41, 42]. The characteristics of our study participants included a majority of multiparas who delivered at term, and from whom one-third had secondary or college education, hence a protective effect on the exposure to obstetric risks.

Consistent with previous studies [42, 43], high CS rate was associated with very low use of LCVE, contrary to the WHO recommendation of appropriate use of emergency obstetric and newborn care (EmONC) services. Further, despite evidence of CS being associated with maternal near miss and death [44], CS was not independently associated with an increased risk of perinatal outcome, and was evenly distributed across the daily shifts. The LCVE rate was less than 2%, consistent with previous studies [42, 43]; and, therefore, in a way confirmed the tendency of obstetricians to avoid difficult obstetric procedures [44]. On the other hand, the low LCVE rate implied an opportunity to improve EmONC.

### Interpretation of the results

Increased prevalence of perinatal morbidity and mortality during off-hours could generally be associated with pre-existing problems in low-resource settings, including delays in reaching and receiving care among referral cases [45, 46]. A high proportion of referred patients in labour during the night hours might have overwhelmed the available resources allocated to the perhaps underestimated number of anticipated night deliveries. This underestimation could either be due to the failure of hospital management to appreciate the daily trend of deliveries or in an attempt to evenly distribute the limited resources; which, in this case, was counterproductive.

The majority of antenatal ward admissions who gave birth during the night shift were presumably pregnant women with poor medical and obstetric histories, as an indication for admission, and hence, they experienced an added recurrent risk of poor perinatal outcome compared to other admissions. On the other hand, some of the admissions might have included low-risk self-referrals, as MNH also received some private patients, although the majority of these came from home. As previously demonstrated [47–50], referrals from other hospitals experienced first-, second- or even third-levels of delay in receiving care at the referring point. The transfer process through the primary and secondary referral facilities could be commonly occurring in the morning and afternoon before the pregnant women eventually reach MNH during the night shift. One can reasonably argue that an increased prevalence of poor perinatal outcome at the highest referral point should be anticipated during the night shift rather than the morning or evening shifts.

The review of the birth registry [32] demonstrated that not all reasons for referral to MNH were due to obstetric complications. The majority of referral indications were non-obstetric, including a lack of sutures, a non-functional theater bed or anaesthetic machine, consumables being out of stock, among others [51, 52]. This highlights that an overwhelmingly increasing workload of deliveries is placed at the highest referral point, without a corresponding increase of resources. The relatively compromised quality of care in terms of human and physical resources during the night compared to the daytime could place increased demand of resources and intensity of work, and hence elevate the level of poor outcomes during off-hours. For example, on-call doctors commence work at 8 am and receive 15 to 40 deliveries per day, among which 30% to 60% are CS, which can reasonably cause severe exhaustion to the care providers in the night shift; and therefore, reduce the quality of the process and outcome of care [53, 25].

We found high rates of CS, which agreed with a previous report [43], which addresses an increasing rate of CS in low-risk groups, and exposed questionable CS indications based on the absence of concomitantly improved neonatal outcomes. Despite a lack of association between CS and poor perinatal outcome in our study, assigning unnecessary CS interventions in resource-limited settings, such as those in Tanzania, indirectly deprives others who are in need of the intervention; hence adding to the risk of poor perinatal outcome. Importantly, a maternal near-miss study on CS at Dar es Salaam public hospitals found CS to be associated with life-threatening events [54], and other studies found delays in the timeline for intervention after taking the decision to perform CS [51, 52], the result of which is that the negative effect might be higher during the night shift when there were more deliveries than during morning or evening shifts, which have less deliveries [32].

Supported by another author [12], our study setting has unlimited access to senior doctors and midwives during the morning shifts compared to off-hours shifts. The benefits of the presence of senior staff during the morning shifts include better workforce and clinical and organizational decision making compared to other shifts, when seniors may be off work. However, the benefits of professional seniority might not necessarily mean better skills in obstetrics and midwifery. Nevertheless, the positive impact of the presence of senior professionals depends on the adequacy of resources and the participation of senior doctors in the daily routine. During this study, senior and experienced nurse midwives were mostly administrators and hence performed less clinical work. Similarly, senior doctors were usually available during the grand rounds, past the 24-h admission report and occasionally during the service morning ward rounds. Senior doctors who were on call attended emergency cases, when called to do so during their 24-h on-call duty. Similar to previous findings [25], the absence of senior doctors during off-hours compromised decision making, hence causing delays or precipitating suboptimal interventions that could have led to poor delivery outcomes. The strengthening of leadership skills during working hours and the off-hours shift, as well as the teamwork skills during the on-call emergency shift, has been found to improve practice [51, 52]. The presence of senior staff and the proportion of working staff per shift positively influences perinatal outcome with some variation from hospital to hospital [53, 25].

High prevalence of perinatal outcome (i.e., 89/1000 deliveries for Neonatal distress, 42/1000 for ENND, and 64/1000 for fresh stillbirth), in a relatively low-risk group of parturients in our study, highlights the overall deficiencies in EmONC [55]. However, the observed independent association of adverse perinatal outcome such as fresh stillbirths with referrals from other hospitals, antenatal admissions and ABD during the night shift stressed a need for special considerations for 24/7 standardization of EmONC within the existing health care system [17].

### Study limitations

Our study had the advantage of having a large sample size, hence it yielded high power. However, as a cross-sectional design we failed to make causal inferences in the highlighted predictors of poor perinatal outcome. A possible bias included an overlapping time of case management from one shift to another, which might have over-expressed adverse perinatal outcome in a particular shift. Caution should be taken in the generalization of hospital-based findings to the general population due to discrepancies in the proportions of the number and mode of deliveries, and in the availability of equipment, supplies and skilled human resources. Importantly, because of the low prevalence of some variables, such as LCVE and ABD, a bias in the comparability of events could be present.

## Conclusion

Off-hours deliveries, particularly during the night shift, were significantly associated with higher proportions of adverse perinatal outcomes, including low Apgar score, early neonatal death and fresh stillbirth, compared to morning and evening shifts. Labour room admissions from antenatal wards, referrals from another hospital and abnormal breech delivery were independent risk factors for poor perinatal outcome, particularly fresh stillbirths. However, absence of poor outcomes based on the time of delivery should be interpreted with caution, as stillbirths may simply be associated with the timing of delivery as opposed to the time of the occurrence of the event. From these findings, we recommend that:

1. The most urgent and cost-effective ways to reduce maternal and perinatal adverse outcome should include upgrading and improving the quality of EmONC services within the existing health care systems, especially in lower referral points, so as to reduce the number of referrals from the national referral hospitals.
2. The hospital administration should appropriately allocate drugs, equipment and supplies according to demand per shift; and re-structure duty rosters to allow for a higher number of staff during off-hours, and the presence of senior and more experienced staff during all 24-h shifts. Working hours for doctors on call should be reduced and perhaps become 12-h on-call shifts instead of 24-h on-call. The restricted working hours will not only reduce staff fatigue but will also motivate them to accept extra workload when necessary, hence improving quality of care.
3. MNH and lower referring hospital should introduce an internal quality assurance system through routine audits, with the purpose of continuously assessing and improving the quality and safety of obstetric interventions including the use of partogram, LCVE and CS.

## Abbreviations

ABD: Abnormal breech delivery
CS: Caesarean section
EmONC: Emergency Obstetric and Neonatal Care
ENND: Early neonatal deaths
IUFD: Intra-uterine fetal death
LCVE: Low cavity vacuum extraction
MMR: Maternal Mortality Ratio
MNH: Muhimbili National Hospital
TDHS: Tanzania Demographic Health Survey
WHO: World Health Organization
